# C57BL/6 and Swiss Webster Mice Display Differences in Mobility, Gliosis, Microcavity Formation and Lesion Volume After Severe Spinal Cord Injury

**DOI:** 10.3389/fncel.2018.00173

**Published:** 2018-06-21

**Authors:** Harun Najib Noristani, Laetitia They, Florence Evelyne Perrin

**Affiliations:** ^1^INSERM U1198, University of Montpellier, EPHE, Montpellier, France; ^2^INSERM U1051, Montpellier, France

**Keywords:** spinal cord injury, glial cells, microcavity, protection, mobility

## Abstract

Spinal cord injuries (SCI) are neuropathologies causing enormous physical and emotional anguish as well as irreversibly disabilities with great socio/economic burdens to our society. The availability of multiple mouse strains is important for studying the underlying pathophysiological response after SCI. Although strain differences have been shown to directly affect spontaneous functional recovery following incomplete SCI, its influence after complete lesion of the spinal cord is unclear. To study the influence of mouse strain on recovery after severe SCI, we first carried out behavioral analyses up to 6 weeks following complete transection of the spinal cord in mice with two different genetic backgrounds namely, C57BL/6 and Swiss Webster. Using immunohistochemistry, we then analyzed glial cell reactivity not only at different time-points after injury but also at different distances from the lesion epicenter. Behavioral assessments using CatWalk™ and open field analyses revealed increased mobility (measured using average speed) and differential forelimb gross sensory response in Swiss Webster compared to C57BL/6 mice after complete transection of the spinal cord. Comprehensive histological assessment revealed elevated microglia/macrophage reactivity and a moderate increase in astrogliosis in Swiss Webster that was associated with reduced microcavity formation and reduced lesion volume after spinal cord transection compared to C57BL/6 mice. Our results thus suggest that increased mobility correlates with enhanced gliosis and better tissue protection after complete transection of the spinal cord.

## Introduction

Spinal cord injury (SCI) is a distressing neuropathology that affects over 2.5 million people worldwide with an annual incidence of 40–60 per million populations (van den Berg et al., 2010). Depending on the anatomical level and lesion severity, clinical symptoms related with SCI range from minor sensory/motor impairments to complete quadriplegia. Following the initial mechanical damages to the spinal cord there are a series of secondary events including neuroinflammation, oedema, microcavity formation and gliosis that altogether govern tissue re-organization and functional impairment after SCI (Hausmann, 2003). Microglia and astrocytes are the two glial cell populations predominantly involved in scar formation.

To uncover the underlying pathophysiology of SCI several animal models have been developed including spinal cord compression, contusion, ischemia, hemisection and complete transection (Lee and Lee, 2013). Recent advances in surgical devices have helped to generate reliable models of SCI in animals with minimal inter-individual variations in lesion severity (Basso et al., 1996b; Onifer et al., 2007). While complete transection is considered as the most deleterious SCI model, it permits to avoid the inter-individual variations in lesion severity (M’Dahoma et al., 2014). In addition, as complete transection model is very reproducible, it allows usage of a smaller number of animals. Furthermore, complete transection is the most rigorous model to study axon regeneration because the two segments of the spinal cord are totally separated with no spared axons (Lee and Lee, 2013). Indeed, complete transection model has been extensively used to study functional recovery, although majority of reports have focused on rats (Basso et al., 1996a; Antri et al., 2005; Onifer et al., 2007; M’Dahoma et al., 2014).

Multiple mouse strains with different transgenic backgrounds are now available to study the underlying pathophysiological mechanisms after SCI. However, there are inherent differences in mouse strains that affects neuroinflammation, tissue preservation and spontaneous functional recovery following incomplete SCI (Basso et al., 2006; Kigerl et al., 2006; Lapointe et al., 2006; Kerr and David, 2007). Previously, Ma et al. (2004) reported reduced inflammation in 129X1-SvJ associated with increase regeneration compared to C57BL/6 mice strain after contusion SCI, although the authors found no difference in functional recovery between the two strains of mice. Subsequently, Kigerl et al. (2006) reported increased microglial/macrophage activation in C57BL/6 after contusion SCI compared to C57BL/10, BALB/c and B10.PL mouse strains, although all four groups displayed similar changes in lesion volume.

To study the specific role of glial cells after SCI, we have recently used transgenic mice that express enhanced green fluorescence protein (eGFP) either in astrocytes (Aldh1l1-EGFP) or in microglia/monocytes (CX3CR1^+/eGFP^; Noristani and Perrin, 2016; Noristani et al., 2016, 2017b). Aldh1l1-EGFP are maintained in Swiss Webster, whereas CX3CR1^+/eGFP^ mice have C57BL/6 genetic background that may influence spontaneous functional recovery and tissue histopathology after spinal cord transection.

Here, we have carried out detailed behavioral and histological assessments after complete transection of the spinal cord in mice with two different genetic background namely, Swiss Webster and C57BL/6, respectively. Using CatWalk™ and open field analyses we observed increased mobility after complete transection in Swiss Webster compared to C57BL/6 mice that was associated with increased gliosis, better tissue preservation and reduced lesion volume. Our findings highlight the positive correlation between increased mobility, enhanced gliosis and better tissue protection after complete transection of the spinal cord.

## Materials and Methods

### Animals

All experiments followed the French and EU regulations (EU/Directive/2010/63 of the European Parliament and Council). All experimental protocols were approved by the “Ministère de l’Education Nationale, de l’Enseignement Supérieur et de la Recherche” and the regional ethic committee for animal experimentation (CEEA-Languedoc Roussillon, authorization number: 34,118), France. All efforts were made to minimize animal number and their suffering. We used, transgenic mice strains expressing selectively enhanced green fluorescent protein (eGFP) either in astrocytes (Aldh1l1-EGFP)OFC789Gsat/Mmucd, Mutant Mouse Regional Resource Centre) (Cahoy et al., 2008) or in microglia (CX3CR1, obtained from Dr. Dan Littman, Howard Hughes Medical Institute, Skirball Institute, NYU Medical Centre, NY, USA). These mice are maintained in different genetic backgrounds namely, Swiss Webster for Aldh1l1 and C57B/L6 for CX3CR1 mice, respectively. Heterozygous CX3CR1^+/eGFP^ and wild type (WT) Aldh1l1 (not expressing eGFP) were used in the current study that were confirmed by polymerase chain reaction (PCR). Previous studies have shown that CX3CR1^+/eGFP^ display similar microglia phenotype to IBA1-EGFP transgenic mice (Hirasawa et al., 2005). Transcriptomic analysis revealed less than 0.5% differences in deregulated genes between WT and CX3CR1^+/eGFP^ microglia (Solga et al., 2015). More recently, Hirbec et al. (2018) also reported no difference in deregulated genes between WT and CX3CR1^+/eGFP^ microglia after lipopolysaccharide challenge. Altogether, these studies suggest that eGFP expression in CX3CR1^+/eGFP^ microglia do not affect their phenotype under both physiological and inflammatory condition. We have also carried out immuohistochemical analysis for GFAP and IBA1 expression following injury in WT and Aldh1l1-EGFP littermates and observed no difference in glial response (data not shown). We will from now refer in the text to mice strains as Swiss Webster and C57BL/6. All mice were housed in controlled conditions (hygrometry, temperature and 12 h light/dark cycle).

### Spinal Cord Injury and Postoperative Cares

Adult female mice (12 weeks) were anesthetized and received inhaled isoflurane gas (1.5%); a thoracic 9 (T9) laminectomy was performed and complete transection was carried out under the microscope (Leica M80, Nanterre, France) using a micro-scalpel (10315-12, Fine Science Tools, Heidelberg, Germany), as described previously (Noristani et al., 2015, 2016, 2017a,b). Table 1 summarizes the number of animals used for behavioral analysis and histological assessment at multiple time-points after injury. Lesions were done at T9 vertebra level to obtain complete paraplegia whilst preserving full respiratory function. Bladders were emptied manually twice daily throughout the study period. Bodyweight was measured before surgery and continued daily till 6 weeks post-lesion.

**Table 1:** Summary of the number of animals used for behavioral analysis and histological assessment at multiple time-points after complete transection of the spinal cord in both strains of mice.

Strain	Behavioral analysis		Histological analysis					Total
	Un-injured	Injured	Un-injured	1 week	2 weeks	4 weeks	6 weeks	79
Swiss Webster	5	5	5	6	6	6	5	38
C57BL/6	6	8	5	6	4	6	6	41

### Behavioral Analysis

Mice underwent behavioral assessments (CatWalk™ and open field) at 7 and 1 days before injury followed by 24 and 72 h and then once a week up to 6 weeks after lesion compared to that of non-injured (NI) control.

#### CatWalk™

CatWalk™ permits the dynamic walking patterns analysis (Noldus, Wageningen, Netherlands). Several parameters (total speed, print area and max contact) were analyzed. We used the CatWalk™ associated with CatMerge (InnovationNet, France), as previously described (Gerber et al., 2012). Eight to ten runs per animal were performed at each time-point with a minimum of five runs at the same speed; at least 3-full step sequence patterns per run were recorded. To avoid bias due to stress, we placed all mice on the CatWalk™ plate for 20 min 7 days prior to the first recording session.

#### Open Field Activity

Spontaneous locomotor activity was monitored using open field test (Supplementary Figure S1B). Mice were placed in an empty test arena (45 × 45 cm box) and their movements were automatically recorded by a camera. Average speed was calculated using EthoTrack (InnovationNet, Tiranges, France). In addition, we also evaluated modification in forelimb gross sensory response after injury using sandpapers, as previously described (Wetzel et al., 2007). The open field floor was divided into four areas composed of four different granularities of sandpapers with mean grain size of 100, 200, 270 and 425 /μm (Supplementary Figure S1B). Considering the mean grain size in each of the four sandpapers, the open field was divided into smooth (mean grain size 100/μm, 1 compartment) and rough (mean grain size >200/μm, three compartments). Times spent on the smooth and rough sandpapers were analyzed automatically using EthoTrack (InnovationNet, Tiranges, France). Mice were placed on individual sandpapers and positioned on the same surface area and orientation. Each analysis session involved 20 min of recording (10 min on plain ground for motor activity followed by 10 min on sandpaper grounds) preceded by 4 min without recording. All mice were placed in the open field for a period of 30 min 7 days prior to the first recording session. To assess SCI-induced anxiety in mouse behavior, the open field data were analyzed by dividing the arena into centralized (22.5 × 22.5 cm) area and the remaining outside zone. Time spent on the centralized and outside zones were measured throughout 6 weeks after injury.

#### Primary Antibodies for Immunological Staining

Primary antibodies used included polyclonal rabbit anti GFAP (1:1000; Z0334; Dako, Glostrup, Denmark), mouse anti vimentin (1:500; V2258–2ML; Sigma Aldrich, St. Louis, USA) and rabbit anti-IBA1 (1:1000; 019–19741; Wako Pure Chemical Industries, Osaka, Japan).

#### Immunohistochemistry

Mice were anesthetized with tribromoethanol (500 mg/kg, i.p) and perfused intracardially with 0.1 M phosphate base saline (PBS) followed by 4% paraformaldehyde (PFA, Sigma Aldrich, St. Louis, USA). Spinal cords were removed and post-fixed for additional 2 h in 4% PFA, cryoprotected in 30% sucrose, included in Tissue Teck (Sakura, Alphen aan den Rijn, Netherlands), frozen and kept at −20°C until processing. Spinal cords were cut longitudinally (22 μm) using a cryostat (Microm, Heidelberg, Germany) and were collected on super frost plus slides. For fluorescence immunohistochemistry, slides were washed in 0.1 M PBS and incubated for 20 min in 20 mM lysine (pH 7.2). Sections were then permeabilized and blocked for 1 h with 0.1 M PBS containing 1% bovine serum albumin (BSA, Sigma Aldrich, St. Louis, USA) and 0.1% Triton X-100 (Fisher Scientific, Illkirch, France) followed by incubation in primary antibody for 48 h at 4°C. Slides were then washed in 0.1 M PBS and were placed in corresponding secondary antibodies conjugated to Alexa 488, 594 or 633 (Vector Laboratories, Peterborough, United Kingdom and Millipore Bioscience Research Re-agents, Fontenay sous Bois, France). Sections were cover slipped using fluorescence mounting medium (Dako, Glostrup, Denmark). Morphometric fluorescent photographs were obtained and analyzed using NanoZoomer RS slide scanner (NanoZoomer Digital Pathology System and NDP view software, Hamamatsu, Japan). In addition, we also used laser scanning inverted (Leica SP5, Mannheim, Germany) and (Zeiss five Live Duo, Oberkochen, Germany) confocal microscopy. Four time-points after injury were analyzed at histological levels namely, 1, 2, 4 and 6 weeks after lesion (Table 1). All sections used for immunofluorescence analysis were selected in random. Sections were number coded to ensure blind analyses (both strain and time post-lesion) throughout the experiment. The codes were not revealed until the end of the experiment.

#### Immunohistochemistry Analysis

For measurement of astrogliosis and microglia/macrophage response three to five sections per mouse were analyzed per time-points. Astrogliosis was assessed using glial fibrillary acidic protein (GFAP) and vimentin staining. For microgliosis we used ionized calcium-binding adapter molecule 1 (IBA1) immunostaining. Five different zones at specific distances away from the edge of the astroglia scar border across the entire diameter of each longitudinal section rostral and caudal to the lesion sites were analyzed (Supplementary Figure S1A). These zones included (Z1) 0–250 μm immediately adjacent to the lesion site, (Z2) between 250–500 μm away, (Z3) 1000–1250 μm away, (Z4) 2000–2250 μm away and (Z5) 4000–4250 μm from the lesion site. Zones across the spinal cord sections were traced in at least *n* = 4 mice per group (4–7 mice per group) using a computer-driven stage. In NI mice, we analyzed optical density and area fraction in the equivalent anatomical levels of the spinal cord (Supplementary Figure S1A).

#### Relative Optical Density

To determine glial reactivity, the mean optical density was measured at different distances and time-points after SCI. Optical density is a sensitive and reliable approach to measure expression level of a given signal and to detect changes caused by experimental conditions. Immunofluorescence labelled spinal cord sections were scanned using Nanozoomer RS slide scanner that uses constant exposure time to obtain fluorescence-labeled photographs (NanoZoomer Digital Pathology System and NDP view software, Hamamatsu, Japan). To further avoid potential variation in staining intensity between different slides or animals, for each of the given antibody, we carried out immunostaining of all animals in parallel. The different zones used for optical density analysis were identified using Nanozoomer software and digital images were exported as RGB using identical exposure settings. Optical density quantification was performed using ImageJ software (NIH, USA). Optical density measures were determined in randomly selected longitudinal section with clearly visible lesion site from each mouse spinal cord. To measure staining density in different zones, optical density measures included the individual zones both rostral and caudal to the lesion site in each section.

#### Area Fraction

To analyze the proportional area occupied by glial cells at different distances to the lesion site, we carried out area fraction analysis using ImageJ software (NIH, USA). All sections used for optical density measurement also underwent area fraction analysis. Threshold was adjusted according to NI control and this was kept constant for all images analyzed. Area fraction was analyzed individually in all five mentioned zones both rostral and caudal to the lesion site.

#### Quantification of Microcavities and Lesion Volume

Microcavities are devoid of reactive astrocytes and are frequently observed in SCI patients and animal models. To identify microcavities, longitudinal sections were stained using GFAP antibody, as described previously (Thuret et al., 2012). Microcavities were measured as the area fraction in Z1–Z5 of tissues without GFAP staining as compared to total surface area, both rostral and caudal to the lesion site. Lesion volume was also measured in GFAP-stained longitudinal sections, as previously described (Nelissen et al., 2014; Gadani et al., 2015). Microcavities and lesion volume were measured in over five longitudinally cut sections per mouse with 110 μm intervals throughout the spinal cord. The area with no GFAP was selected as lesion area and was measured in each animal of the group (*n* = 4–8). Lesion area in each section was quantified using ImageJ and lesion volume was quantified by multiplying the measured surface area by the section thickness. Measured volumes were then summed for the final estimation of lesion volume in cubic millimeters.

#### Statistical Analysis

Two-way ANOVA with Bonferroni *post hoc* tests were used for multiple comparisons in the two strains of mice (behavior, GFAP, IBA1 and microcavities). One-way ANOVA with Tukey *post hoc* tests were used for multiple comparisons (staining intensities at different distance to injury vs. NI control) for each given mouse strain. To directly compare the two strains, we first verified that the basal level for a given parameter (basal behavioral parameter, protein intensity and microcavities percentage) in NI animals were similar in both strains of mice. GFAP expression and cavities were similar in both strains of mice prior to injury. For behavioral parameters and IBA1 intensity, as basal expression levels were different between the two strains in un-injured controls, we normalized the data for each given strain to the value obtained before injury (behavior parameters) or to NI controls (IBA1 intensity), respectively. Un-paired *t*-tests were used to compare differences in lesion volume. Significance was accepted at *p* ≤ 0.05. All data were analyzed using GraphPad Prism 4.0 (GraphPad Software, Inc., CA, USA). Data are shown as the mean ± standard error of the mean (SEM).

## Results

### Swiss Webster Show Increased Mobility Than C57BL/6 Mice After Complete Transection

**Figure 1:**
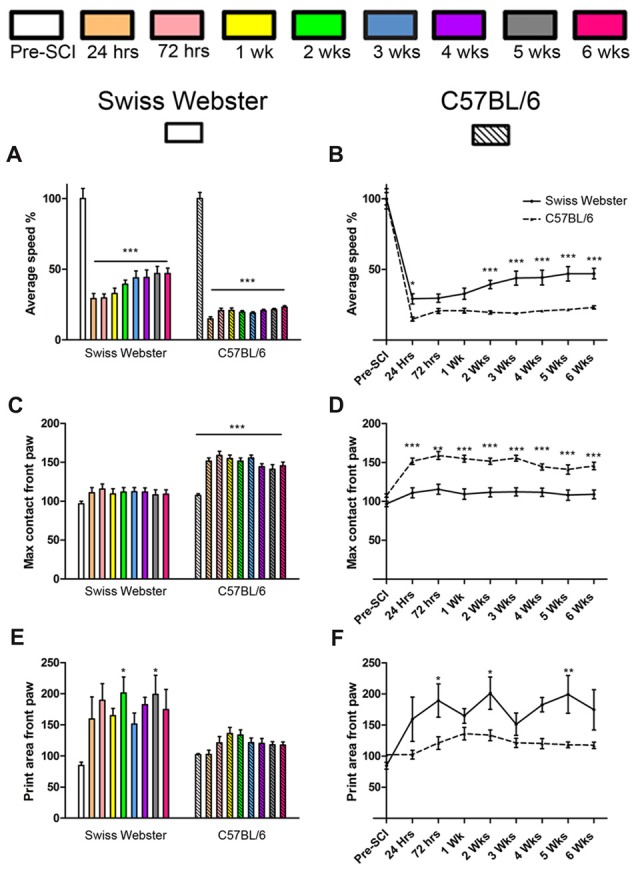
Swiss Webster display increased mobility after spinal cord transection as compared to C57BL/6 mice. Bar and line graphs displaying alterations in average speed using CatWalk™ analysis after spinal cord transection in Swiss Webster and C57BL/6 mice **(A,B)**. Swiss Webster displayed increase average speed **(A,B)**, no change in max contact **(C,D)** and augmented usage of their front paws (print area; **E,F**) compared to C57BL/6 mice. Conversely to Swiss Webster, C57BL/6 mice displayed increased max contact throughout the 6 weeks **(C,D)**. One-way ANOVA with Tukey *post hoc* tests **(A,C,E)** and two-way ANOVA with Bonferroni *post hoc* **(B,D,F)**. *P < 0.05, **P < 0.01, ***P < 0.001.

To determine strain-dependent differences in spontaneous functional recovery post-injury, we carried out detailed behavioral assessments using CatWalk™ and open field analyses. Analysis of average speed using CatWalk™ revealed pronounced decrease in mobility after injury in both strains of mice at 24 h post-lesion reaching 29% in the case of Swiss Webster and only 15% for C57BL/6 mice compared to before surgery (Figures 1A,B). Swiss Webster mice displayed a steady increase in their average speed overtime reaching over 47% by 5 weeks post-lesion compared to before injury (Figures 1A,B). C57BL/6 mice, on the other hand, did not display increased speed overtime, they indeed remained at 23% at 6 weeks post-lesion compared to before injury (Figures 1A,B). Thus, Swiss Webster mice showed a less affected speed and a better recovery after injury as compared to C57BL/6 mice throughout the 6 weeks period after spinal cord transection (Figures 1A,B). Increased mobility in Swiss Webster mice after complete transection was also evident using max contact parameter of the CatWalk™, which is the time a paw contacts the glass plate. Indeed, Swiss Webster did not show an increase in max contact of their front paw whilst C57BL/6 mice displayed an increase in max area from 24 h following injury that remain stable throughout 6 weeks post-injury (Figures 1C,D). Increased speed in Swiss Webster mice was due to greater function of their front paws as indicated by an increase in print surface area compared to C57BL/6 mice (Figures 1E,F). No movement of the hind paws was observed in either Swiss Webster or C57BL/6 mice throughout 6 weeks analyses after spinal cord transection (Supplementary Figures S1C,D). Following complete transection of the spinal cord we only observed dragging of the hind paws. No plantar placement of the hind paws was evident in either strains of mice throughout 6 weeks after lesion.

**Figure 2:**
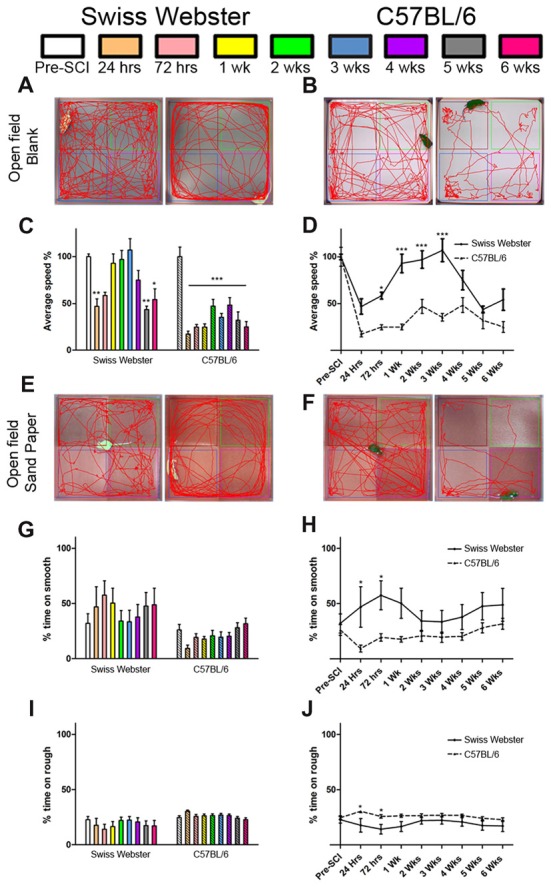
Swiss Webster display increased mobility and differential gross sensory response after spinal cord transection as compared to C57BL/6 mice. Photomicrographs indicating the experimental arrangement of the open field test used to measure average speed after spinal cord transection in Swiss Webster **(A)** and C57BL/6 mice **(B)**. Bar and line graphs displaying alterations in average speed using open field analysis after spinal cord injuries (SCI) in Swiss Webster and C57BL/6 mice **(C,D)**. Swiss Webster displayed increased average speed compared to C57BL/6 mice **(D)**. Photomicrographs indicating the experimental arrangement of the sandpaper test used to measure sensory response after spinal cord transection **(E,F)**. Bar and line graphs displaying alteration in sensory response after SCI in Swiss Webster and C57BL/6 mice **(G–J)**. Neither Swiss Webster mice nor C57BL/6 mice displayed change in their sensory response over the course of their behavioral follow-ups, as indicated by similar interest in both smooth and rough sandpapers throughout the 6 weeks **(G,I)**. However, direct comparison between the two strains revealed a moderate preference of Swiss Webster mice for the smooth area and a preference of the rough area in C57BL/6 at 24 and 72 h post-lesion. One-way ANOVA with Tukey *post hoc* tests **(C,G,I)** and two-way ANOVA with Bonferroni *post hoc* **(D,H,J)**. *P < 0.05, **P < 0.01, ***P < 0.001.

Open field analysis further confirmed increased mobility in Swiss Webster than C57BL/6 mice after spinal cord transection (Figure 2). Swiss Webster mice displayed significant decrease in their average speed up to 72 h post-lesion followed by a transient increase in speed between 1–3 weeks post-lesion (Figures 2A,C). Conversely, C57BL/6 mice displayed constant reduced speed throughout 6 weeks after injury compared to before surgery (Figures 2B,C). Direct comparison between the two strains of mice using open field showed significant increase in speed from 72 h until 3 weeks after SCI in Swiss Webster compared to C57BL/6 mice (Figure 2D). To determine whether the increased mobility reflects differences in anxiety behavior, we analyzed the open field recordings for anxiety (Supplementary Figure S2). SCI induced a pronounced increase in anxiety behavior as indicated by reduced time spent in the center of the open field in the two stains of mice. Direct comparisons revealed no significant difference in anxiety behavior between Swiss Webster and C57BL/6 mice after complete transection of the spinal cord (Supplementary Figure S2). Taken together, these data demonstrate that Swiss Webster show better anxiety-independent increase in mobility than C57BL/6 mice after complete transection of the spinal cord.

### Swiss Webster Show Differential Forelimb Gross Sensory Response After Spinal Cord Transection

Next, to investigate possible modification in mouse forelimb sensory tactile response after spinal cord transection, we used open field consisting of sandpapers with low (smooth) and high (rough) granularities, as described previously (Wetzel et al., 2007). C57BL/6 mice showed preference for the rough sandpaper whilst Swiss Webster mice displayed preference for the smooth sandpaper, as reflected by increase in time spent in these areas of the open field (Figures 2E,F,G,I). Such minor preferences for the rough and smooth sandpapers were evident early after lesion and did show significant difference compared to before injury (Figures 2G,I). However, direct comparison between the two strains of mice revealed significant differences in their preference between the rough and smooth sandpapers at 24 and 72 h post-lesion (Figures 2H,J). Altogether, tests using sandpapers with different granularities demonstrated differential forelimb gross sensory response between Swiss Webster and C57BL/6 mice after complete transection of the spinal cord.

### Swiss Webster Display Increased Gliosis Than C57BL/6 Mice After Severe SCI

**Figure 3:**
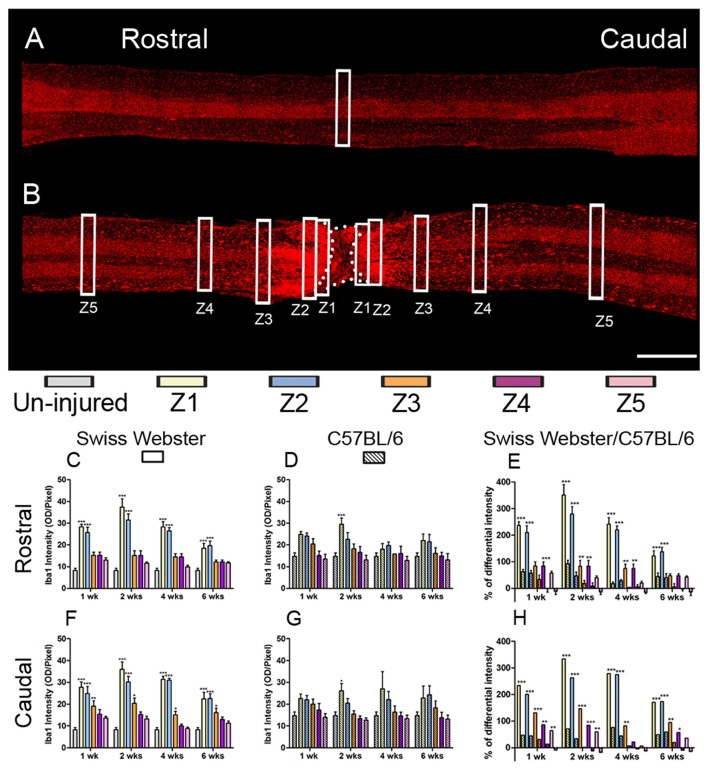
Increased microglia/macrophage response in Swiss Webster compared to C57BL/6 mice after severe SCI. Confocal photomicrographs indicating IBA1 staining of longitudinal spinal cord sections in the non-injured (NI) control and spinal cord transected mice **(A,B)**. Bar graphs indicating quantitative analysis of IBA1 immunoreactivity along the five zones rostral and caudal to the lesion site in Swiss Webster mice **(C,F)** and C57BL/6 mice **(D,G)**. Direct comparisons following normalization revealed that Swiss Webster displayed increased IBA1 immunoreactivity compared to C57BL/6 mice after spinal cord transection **(E,H)**. Scale bars **(A,B)**: 1 mm. One-way ANOVA with Tukey *post hoc* tests **(C,D,F,G)** and two-way ANOVA with Bonferroni *post hoc* **(E,H)**. *P < 0.05, **P < 0.01, ***P < 0.001.

**Figure 4:**
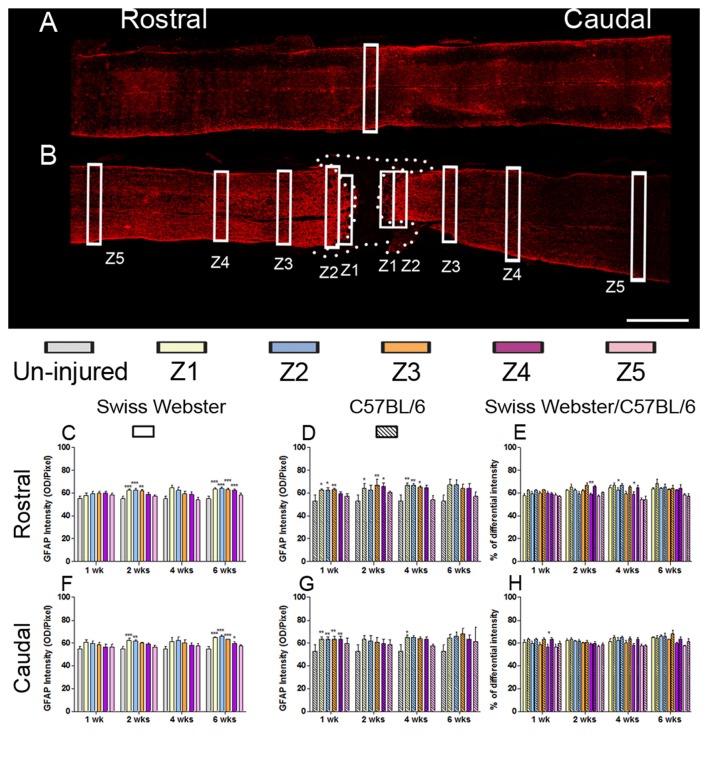
Astrogliosis in Swiss Webster and C57BL/6 after severe SCI. Confocal photomicrographs indicating GFAP staining of longitudinal spinal cord sections in the NI control and spinal cord transected mice **(A,B)**. Bar graphs indicating quantitative analysis of GFAP immunoreactivity along the five zones rostral and caudal to the lesion site in Swiss Webster mice **(C,F)** and C57BL/6 mice **(D,G)**. Direct comparisons revealed that C57BL/6 mice displayed moderate increase in GFAP immunoreactivity compared to Swiss Webster after complete transection of the spinal cord **(E,H)**. Scale bars **(A,B)**: 1 mm. One-way ANOVA with Tukey *post hoc* tests **(C,D,F,G)** and two-way ANOVA with Bonferroni *post hoc* **(E,H)**. *P < 0.05, **P < 0.01, ***P < 0.001.

To examine the potential correlation between increased mobility and glial cell reactivity after severe SCI, we next analyzed microglia/macrophage and astrocyte reactivity between Swiss Webster and C57BL/6 mice following complete transection of the spinal cord. We quantified IBA1 and GFAP intensities and area fraction in five different zones located at specific distances away from the edge of the astroglia scar border including: (Z1) 0–250 μm immediately adjacent to the lesion site, (Z2) between 250–500 μm away, (Z3) 1000–1250 μm away, (Z4) 2000–2500 μm away and (Z5) 4000–4250 μm from the lesion site, as previously described (Wanner et al., 2013; see also Figures 3, 4 and Supplementary Figure S1A). In general, gliosis was similar between rostral and caudal to the lesion site in both strains of mice (Figure 3; Swiss Webster Figures 3C,F and C57BL/6 Figures 3D,G). Increased gliosis was more evident within Z1–Z3 compared to more distal regions (Figures 3, 4).

Specifically, Swiss Webster displayed a widespread increase in IBA1 intensity on both sides of the lesion expanding up to zone two that was evident till 6 weeks after injury (Figures 3C,F). C57BL/6 mice, on the other hand, displayed a limited increase in IBA1 intensity only in Z1 at 2 weeks after traumatism rostral and caudal to the lesion site (Figures 3D,G). Comparisons between the two strains of mice revealed a clear increase in IBA1 intensity in Swiss Webster compared to C57BL/6 mice after spinal cord transection (Figures 3E,H). Analysis of area fraction stained with IBA1 further confirmed a higher activation of macrophage/microglia in the Swiss Webster strain as compared to C57BL/6 (Supplementary Figures S3A–F).

GFAP expression in Swiss Webster mice was increased on both sides of the lesion (Z1–Z3) at 2 and 6 weeks post-injury (Figures 4C,F), whereas in C57BL/6 mice GFAP expression was increased up to 4 weeks post-injury in Z1–Z3 rostral to the lesion site and only up to 1 week after traumatism caudal to the injury site (Figures 4D,G). Direct comparison between the two strains revealed brief increase in GFAP intensity in C57BL/6 up to 4 weeks rostral to the lesion site (Figures 4E,H) as compared to Swiss Webster. However, analysis of area fraction stained with GFAP identified a moderate increase in astrocyte in Swiss Webster as compared to C57BL/6 strain caudal to the lesion (Supplementary Figure S3L). Swiss Webster mice displayed increase in GFAP area fraction rostro-caudal to the lesion site up to 6 weeks post-lesion, whereas C57BL/6 mice showed no changes in GFAP area fraction caudal to the lesion (Supplementary Figures S3G–K). Within the lesion site, an equal astrogliosis was observed in Swiss Webster and C57BL/6 mice using vimentin (another classical astrocytic marker; Supplementary Figure S4). Altogether, these data demonstrate pronounced microglia/macrophage reactivity as well a moderately increased astrogliosis in Swiss Webster compared to C57BL/6 mice after spinal cord transection.

### Swiss Webster Show Reduced Microcavity and Lesion Volume Than C57BL/6 Mice After Spinal Cord Transection

**Figure 5:**
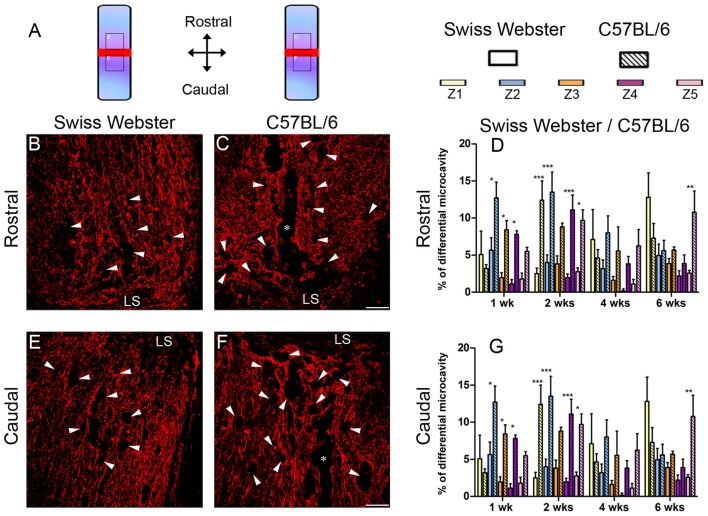
Reduced microcavity formation in Swiss Webster compared to C57BL/6 mice after severe SCI. Schematic drawing of the longitudinal spinal cord sections after complete SCI illustrating the lesion site (red rectangle) and the reference frames for displayed fields of view **(A)**. Confocal photomicrographs of longitudinal spinal cord sections indicating GFAP staining and microcavities (arrowheads) adjacent to the lesion epicenter in Swiss Webster **(B,E)** and C57BL/6 **(C,F)** mice. Bar graphs showing quantitative analysis of microcavities along the five zones rostral **(D)** and caudal **(G)** to the lesion site. Spinal cord transected Swiss Webster displayed reduced microcavities compared to C57BL/6 mice **(C,F)**. Scale bars **(B,C,E,F)**: 100 μm. Two-way ANOVA with Bonferroni *post hoc* tests *P < 0.05, **P < 0.01, ***P < 0.001. LS: lesion site.

**Figure 6:**
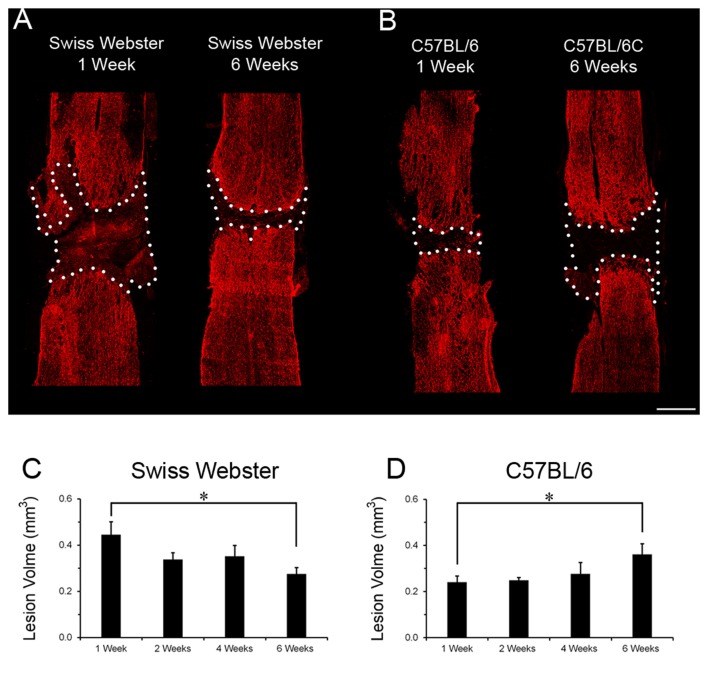
Lesion volume evolution in Swiss Webster and C57BL/6 after severe SCI. Confocal photomicrographs indicating GFAP staining and lesion volume evolution (dotted area) in Swiss Webster and C57BL/6 mice **(A,B)** following complete transection of the spinal cord. Bar graphs showing quantitative analysis of lesion volume evolution over-time in Swiss Webster and C57BL/6 mice after complete SCI **(C,D)**. Swiss Webster displayed time-dependent decrease, whereas C57BL/6 mice showed increased lesion volume between 1 and 6 weeks after complete transection of the spinal cord **(C,D)**. Scale bars **(A,B)**: 500 μm. Un-paired *t*-test *P < 0.05 compared to the same strain at 1 week after injury.

Finally, to examine the relation between strain-dependent differences in gliosis and tissue preservation after severe SCI, we measured the differences in microcavity formation and lesion volume in Swiss Webster and C57BL/6 mice after complete transection of the spinal cord. Microcavities were measured in the five zones (Z1–Z5) both rostral and caudal to the lesion site (Figure 5). In general, microcavities were mostly located within Z1 and Z2 (Figures 5B–F). In both strains, microcavities appeared from 1 week and persisted until 6 weeks following injury. NI animals showed no microcavity (data not shown). Direct comparisons between the two strains of mice revealed significantly reduced microcavities in Swiss Webster compared to C57BL/6 mice after spinal cord transection (Figures 5D,G). Reduced microcavities in Swiss Webster compared to C57BL/6 were evident both rostral and caudal to the injury site and reached significance at 1, 2 and 6 weeks following lesion (Figures 5D,G). Lastly, we measured differences in lesion volume evolution after complete transection of the spinal cord between Swiss Webster and C57BL/6 mice (Figure 6). Quantification of the lesion volume based on GFAP delineation revealed time-dependent reduction in Swiss Webster with significantly decrease between 1 and 6 weeks post-injury (Figures 6A,C). Conversely, C57BL/6 mice displayed time-dependent increase in lesion volume that also reached significance between 1 and 6 weeks after injury (Figures 6B,D). Taken together, these data demonstrate that Swiss Webster mice display reduced microcavity formation and lesion volume than C57BL/6 mice after severe SCI.

## Discussion

In the present study, we carried out detailed behavioral and histopathological analyses after severe SCI in two strains of mice (Swiss Webster and C57BL/6, respectively). Swiss Webster mice displayed increased mobility and differential gross sensory response of their forelimbs following complete transection of the spinal cord than C57BL/6 mice. This behavioral differences post-SCI were associated with enhanced gliosis, reduced microcavity formation and smaller lesion volume. These findings demonstrate strain-dependent differences in behavior after severe SCI that coincides with increased gliosis and preserved histopathology.

### Strain-Dependent Differences in Functional Recovery After SCI

We demonstrate that increased mobility in mice with Swiss Webster genetic background as compared to C57BL/6 mice after complete transection of the spinal cord is associated with increased gliosis, reduced microcavity formation and smaller lesion volume. Others also reported strain-dependent differences in neuroinflammation, tissue preservation and spontaneous functional recovery after incomplete SCI (Basso et al., 2006; Lapointe et al., 2006; Kerr and David, 2007). Inter-strain differences in SCI-induced microglia/macrophages reactivity and neuroinflammation had been reported between C57BL/6, BALB/c and 129X1/SvJ mice after contusion SCI (Ma et al., 2004; Kigerl et al., 2006). However, contrary to our findings, Kigerl et al. (2006) found no consistent correlation between SCI-induced microglia/macrophage activation and lesion size/length in C57BL/6, C57BL/10, BALB/c and B10.PL strains of mice. In fact, the author reported a positive association between increased microglia/macrophage activation and enlarged lesion size only in C57BL/6 mouse strain. Similarly, Ma et al. (2004) had shown that reduced inflammation in 129X1-SvJ mice coincides with increased regeneration compared to C57BL/6 mice after SCI. Divergent to the present study, the two earlier reports applied contusion SCI model that may explain these disparate findings. Previous studies highlight different molecular response including blood-spinal cord barrier (BSCB) leakage between complete transection and other SCI models. Complete transection of the spinal cord at T10 level in institute of cancer research (ICR) stain of mice caused restricted disruption of BSCB than compression SCI, which spread up to cervical level (Pan and Kastin, 2001). In addition, BSCB leakage was only evident within 24 h after complete transection, whereas compression SCI induced a prolonged leakage up to at least 5 days post-lesion (Pan and Kastin, 2001). Another study in C57BL/6 mouse strain also revealed widespread BSCB leakage up to 6 mm rostro-caudal to the lesion that persisted up to 2 weeks after T8 contusion SCI (Whetstone et al., 2003). Altogether, these findings suggest that complete transection mouse model of SCI induces restricted and a relatively short-term BSCB disruption compared to compression and contusion injuries. This in turn limits tissue oedema, neutrophil infiltration, neuroinflammation and subsequent functional outcome after SCI. Although complete transection of the spinal cord is the most deleterious SCI model, it has several advantages including high reproducibility and reduced inter-individual variations in lesion severity (Lee and Lee, 2013; M’Dahoma et al., 2014). Our result provides the first positive correlation between increased mobility and elevated gliosis after complete transection of the spinal cord in Swiss Webster compared to C57BL/6 mice.

### Reactive Microglia After SCI

Microglia, the native immune occupants of the central nervous system (CNS), are the first responsive glial population after SCI (Tian et al., 2007). Activated microglia have a dual role in SCI pathophysiology. In one hand, increased microglial activation promotes inflammation and tissue damage after SCI (David and Kroner, 2011), whilst on the other hand, they express neurotrophic factors (Lalancette-Hébert et al., 2007; Lambertsen et al., 2009) that promote regeneration following SCI (Mukaino et al., 2010). Transplantation of activated microglia/macrophages into the lesion site promotes axonal regeneration in rats after both incomplete (Prewitt et al., 1997; Rabchevsky and Streit, 1997) and complete SCI (Rapalino et al., 1998). In the latter, axonal re-growth was also associated with partial hind limb motor recovery. Microglia/macrophages activation via pre-incubation with lipopolysaccharide favors expression of neurotrophic factors resulting in reduced microcavity formation and improved functional recovery in rats after SCI (Guth et al., 1994; Hashimoto et al., 2005; Hayakawa et al., 2014). Contrary, several reports have shown that treatment with anti-inflammatory agents including methylprednisolone or interleukin 10 reduce microcavity formation and lesion volume associated with improved functional recovery in rats after incomplete SCI (Bethea et al., 1999; Brewer et al., 1999; Takami et al., 2002). Using cell-specific RNA-sequencing, we have recently demonstrated that microglia/macrophages response exclusively depends on time post-SCI, irrespective of lesion severity (Noristani et al., 2017b). Specifically, early after SCI microglia/macrophages proliferate followed by their prominent role in orchestrating neuroinflammation at more chronic stages after both moderate and severe SCI (Noristani et al., 2017b). Interestingly, we have also recently demonstrated differential morphological changes in microglia/macrophages rostro-caudal to the lesion site following complete transection of the spinal cord in mice with C57BL/6 genetic background (Noristani et al., 2017a). Future studies to analyze strain-dependent differences in microglia/macrophages molecular response after SCI awaits further investigations.

### Reactive Astrocytes After SCI

Cavities and necrotic lesion areas are devoid of reactive astrocytes and are commonly observed in both SCI patients and animal models. Earlier qualitative studies reported elevated GFAP immunoreactivity in 129X1-SvJ compared to C57BL/6 mice after contusion SCI (Ma et al., 2004; Dixon et al., 2012), although no differences in functional recovery were found between the two strains of mice (Ma et al., 2004). Our finding of increased astrogliosis after complete SCI, even if limited as compared to microglia/macrophage reactivity, suggest that increased astrocytic scar formation may contribute to limiting cavity formation and lesion expansion in Swiss Webster as compared to C57BL/6 mice. Our data are in line with other studies suggesting the positive role of reactive astrocytes after SCI including reduced microcavity formation (Faulkner et al., 2004), isolation of lesion-associated inflammation (Wanner et al., 2013) and expression of pro-regenerative factors (Anderson et al., 2016). In contrast, other reports suggest a detrimental role of astrogliosis following SCI (Menet et al., 2003; Do Thi et al., 2004; Desclaux et al., 2015; Hara et al., 2017). Recently, using cell-specific RNA-sequencing we have demonstrated that astrocyte response after SCI in Swiss Webster mice depends on both time after injury and lesion severity (Noristani and Perrin, 2016; Noristani et al., 2016). Further studies on molecular response of astrocytes between different strains of mice are necessary to uncover the precise mechanisms responsible for improved functional recovery following SCI.

### Increased Gliosis as a Protective Mechanism After Severe SCI

Our findings suggest that increased mobility correlates with increased gliosis, reduced microcavity formation and smaller lesion volume in Swiss Webster than C57BL/6 mice after complete SCI. It is important to note that increased mobility was due to greater use of front paws since we observed no movement of the hind limbs in either Swiss Webster or C57BL/6 mice throughout 6 weeks analyses. Equally important, all mice showed complete anesthesia in their dermatomes caudal to the injury. Our assessment of gross sensory response using sandpapers with different granularities is primarily due to sensation in the forelimbs. This suggests that the increased mobility coincides with local changes in the spinal cord, rather than possible regrowth and reconnection of few descending spinal tracts across the lesion site. In particular, reduced microcavities and smaller lesion volume in Swiss Webster mice are likely to have contributed to the increased mobility. Interestingly, an earlier report in rats with complete transection of the spinal cord had demonstrated that physical exercise prevents regressive changes in the motor neurons and dendrites (Gazula et al., 2004). Given the increased mobility in Swiss Webster, such augmented exercise may also participate in better tissue preservation than C57BL/6 mice after severe SCI. Further studies are required to uncover the specific basis for the increased mobility in Swiss Webster mice after complete transection of the spinal cord.

## Conclusion

In conclusion, Swiss Webster and C57BL/6 mice were subjected to complete spinal cord transection to study mobility, gliosis and secondary damages after severe SCI in two strains of mice. Increased mobility was observed in Swiss Webster that coincides with increased gliosis, reduced microcavity formation and condensed lesion volume compared to C57BL/6 after complete SCI. Uncovering the molecular mechanisms responsible for glial protection may provide novel therapeutic strategies to minimize secondary damages after severe SCI.

## Author Contributions

HNN conceptualized the research, designed the project, performed majority of the experiments, analyzed the data and contributed to the writing of the manuscript. LT participated in histological experiments and analysis. FEP conceptualized the research, designed the project, participated in the analysis and data interpretation, drafted the work and gave final approval.

## Conflict of Interest Statement

The authors declare that the research was conducted in the absence of any commercial or financial relationships that could be construed as a potential conflict of interest.

## Supplementary Material

FIGURE S1 Schematic drawing of the longitudinal spinal cord sections from non-injured (NI) control and after complete spinal cord injury (SCI) illustrating the lesion site (red rectangle) and five zones (Z1–Z5) of 250 μm width used for measuring glial reactivity **(A)**. Photomicrographs indicating the experimental arrangement of open field and sandpaper tests used to measure average speed and sensory response after SCI, respectively **(B)**. Photomicrographs displaying example of a full step cycle in NI control **(C)** and spinal cord transected mice **(D)**. Note that no movement of the hind paws were noted in either Swiss Webster or C57BL/6 mice after complete transection of the spinal cord throughout 6 weeks post-injury.

FIGURE S2 Swiss Webster and C57BL/6 mice display similar anxiety behavior after spinal cord transection. Bar graphs displaying alterations in anxiety behavior after complete transection of the spinal cord in Swiss Webster **(A)** and C57BL/6 mice **(B)**. Both Swiss Webster and C57BL/6 mice displayed similar increase in anxiety behavior after SCI throughout 6 weeks analysis. One-way ANOVA with Tukey *post hoc* tests. ****P* < 0.001 compared to before surgery.

FIGURE S3 Differential glial response in Swiss Webster and C57BL/6 mice after severe SCI. Bar graphs indicating quantitative analysis of IBA1 area fraction along the five zones rostral and caudal to the lesion site in Swiss Webster **(A,D)** and C57BL/6 mice **(B,E)**. Direct comparisons revealed that spinal cord transected Swiss Webster displayed increased IBA1 area fraction compared to C57BL/6 mice both rostral and caudal to the lesion site **(C,F)**. Bar graphs indicating quantitative analysis of GFAP area fraction along the five zones rostral and caudal to the lesion site in Swiss Webster **(G,J)** and C57BL/6 mice **(H,K)**. Direct comparison showed that transected Swiss Webster displayed moderate increase in GFAP area fraction compared to C57BL/6 caudal to the lesion site **(L)**. One-way ANOVA with Tukey *post hoc* tests **(A,B,D,E,G,H,J,K)** and two-way ANOVA with Bonferroni *post hoc* **(C,F,I,L)**. **P* < 0.05, ***P* < 0.01, ****P* < 0.001.

FIGURE S4 Astrogliosis within the lesion site in Swiss Webster and C57BL/6 mice after severe SCI. Confocal photomicrographs indicating vimentin staining in the NI control and transected longitudinal spinal cord sections **(A,B)**. Bar graphs indicating quantitative analysis of vimentin immunoreactivity at different stages after spinal cord transection **(C,D)**. Transected Swiss Webster and C57BL/6 mice displayed similar increase in vimentin immunoreactivity **(C,D)**. One-way ANOVA with Tukey *post hoc* tests. ***P* < 0.01, ****P* < 0.001. Scale bars **(A,B)**: 1 mm.
