# Supplementary figures and images for: C57BL/6 and Swiss Webster Mice Display Differences in Mobility, Gliosis, Microcavity Formation and Lesion Volume After Severe Spinal Cord Injury
Source: Front Cell Neurosci. 2018 Jun 21;12:173. doi: 10.3389/fncel.2018.00173 (PMC6021489; doi:10.3389/fncel.2018.00173)

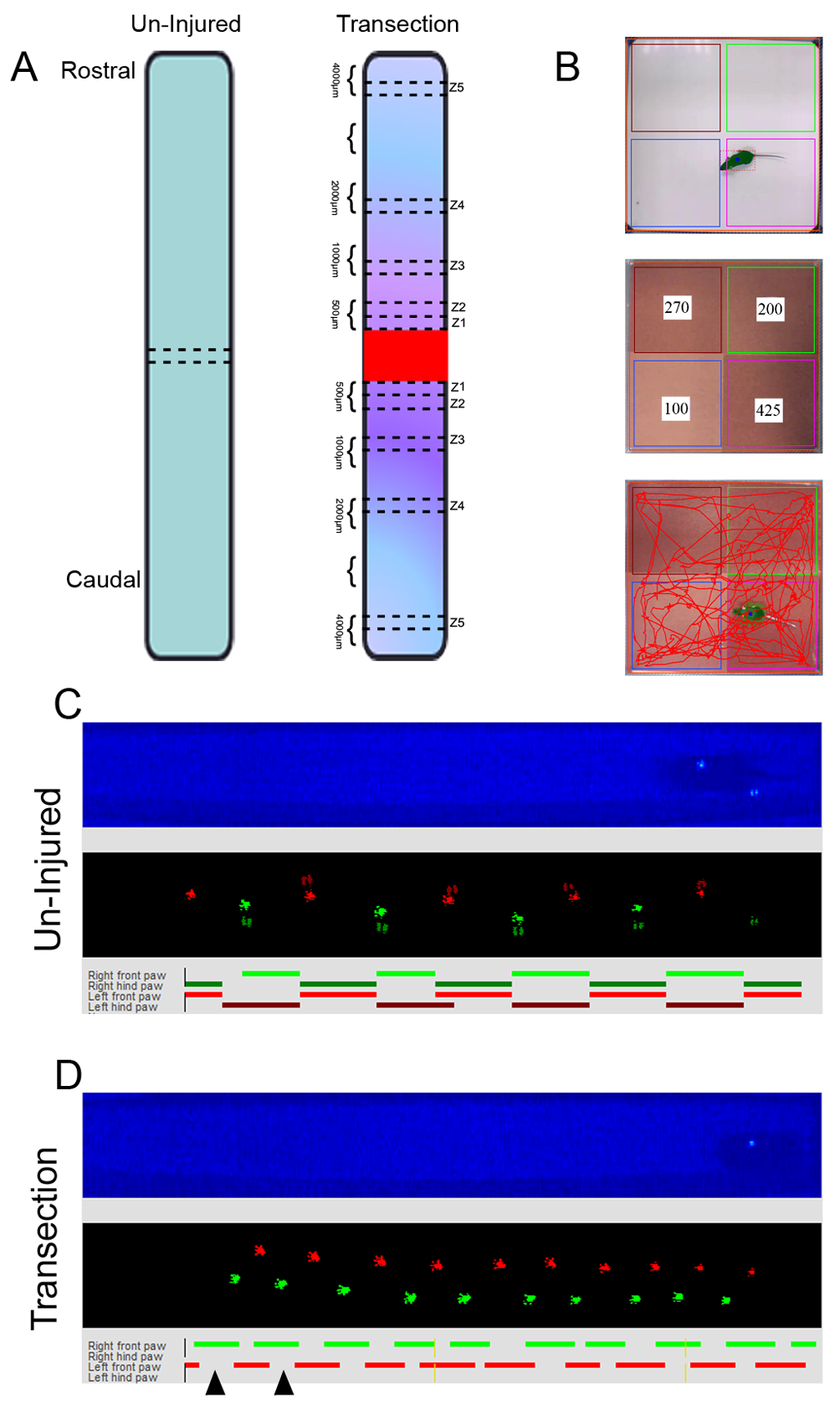

Supplement: FIGURE S1 — Schematic drawing of the longitudinal spinal cord sections from non-injured (NI) control and after complete spinal cord injury (SCI) illustrating the lesion site (red rectangle) and five zones (Z1–Z5) of 250 μm width used for measuring glial reactivity (A). Photomicrographs indicating the experimental arrangement of open field and sandpaper tests used to measure average speed and sensory response after SCI, respectively (B). Photomicrographs displaying example of a full step cycle in NI control (C) and spinal cord transected mice (D). Note that no movement of the hind paws were noted in either Swiss Webster or C57BL/6 mice after complete transection of the spinal cord throughout 6 weeks post-injury. [file Image_1.TIF]

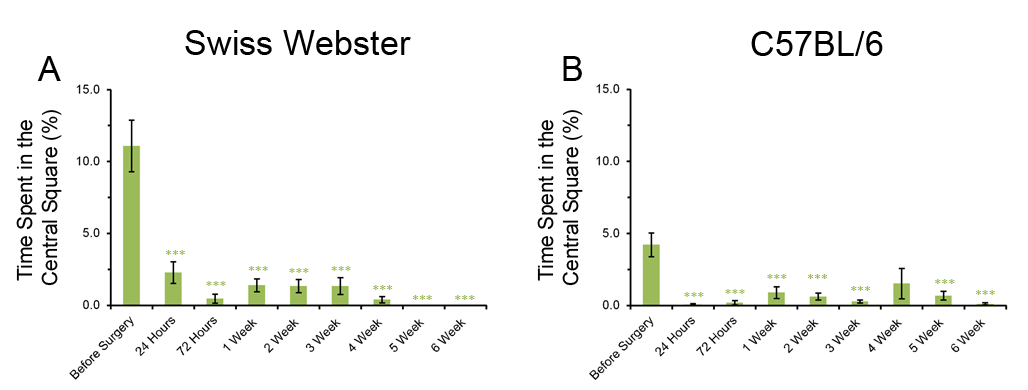

Supplement: FIGURE S2 — Swiss Webster and C57BL/6 mice display similar anxiety behavior after spinal cord transection. Bar graphs displaying alterations in anxiety behavior after complete transection of the spinal cord in Swiss Webster (A) and C57BL/6 mice (B). Both Swiss Webster and C57BL/6 mice displayed similar increase in anxiety behavior after SCI throughout 6 weeks analysis. One-way ANOVA with Tukey post hoc tests. ***P < 0.001 compared to before surgery. [file Image_2.TIF]

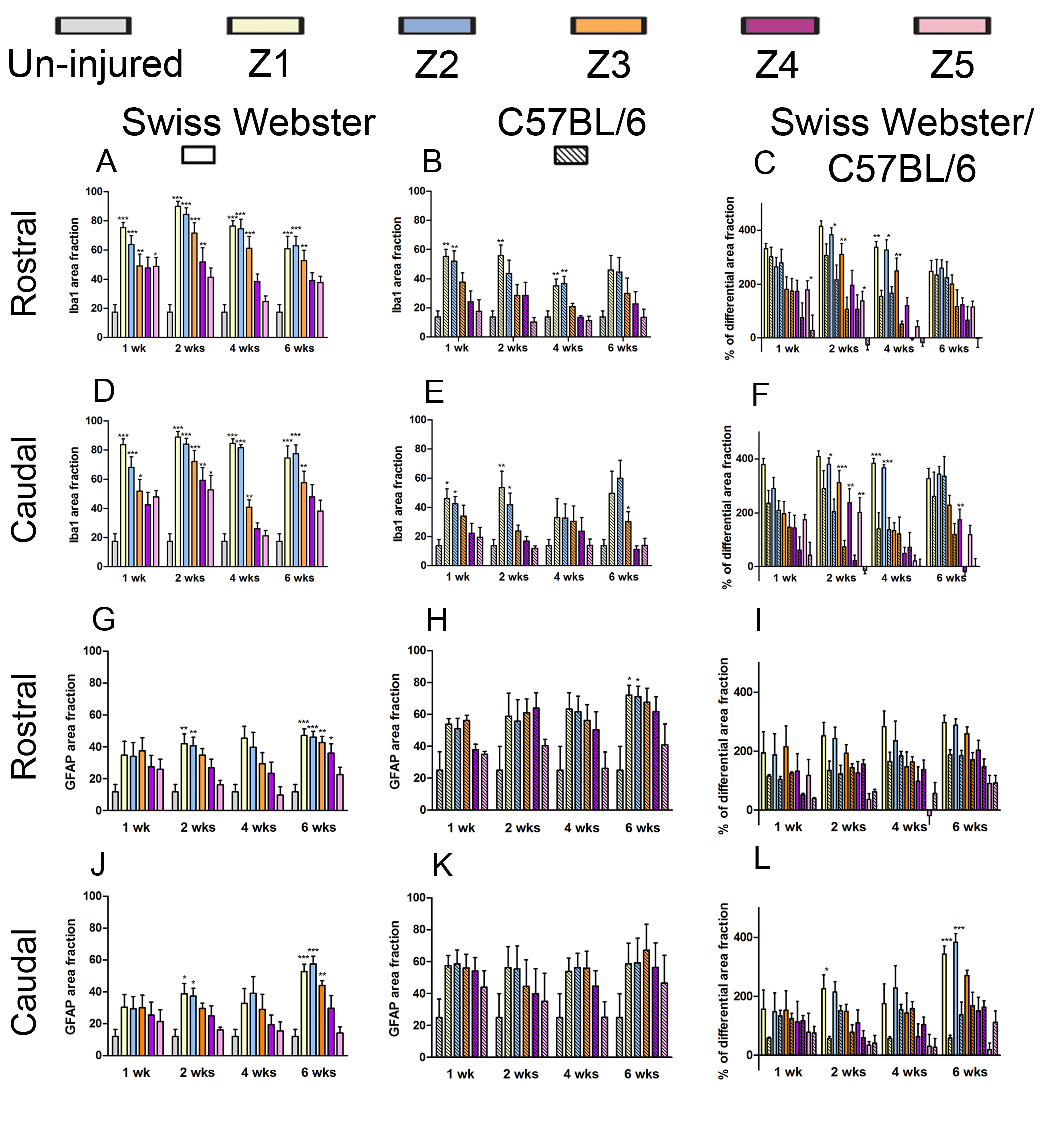

Supplement: FIGURE S3 — Differential glial response in Swiss Webster and C57BL/6 mice after severe SCI. Bar graphs indicating quantitative analysis of IBA1 area fraction along the five zones rostral and caudal to the lesion site in Swiss Webster (A,D) and C57BL/6 mice (B,E). Direct comparisons revealed that spinal cord transected Swiss Webster displayed increased IBA1 area fraction compared to C57BL/6 mice both rostral and caudal to the lesion site (C,F). Bar graphs indicating quantitative analysis of GFAP area fraction along the five zones rostral and caudal to the lesion site in Swiss Webster (G,J) and C57BL/6 mice (H,K). Direct comparison showed that transected Swiss Webster displayed moderate increase in GFAP area fraction compared to C57BL/6 caudal to the lesion site (L). One-way ANOVA with Tukey post hoc tests (A,B,D,E,G,H,J,K) and two-way ANOVA with Bonferroni post hoc (C,F,I,L). *P < 0.05, **P < 0.01, ***P < 0.001. [file Image_3.TIF]

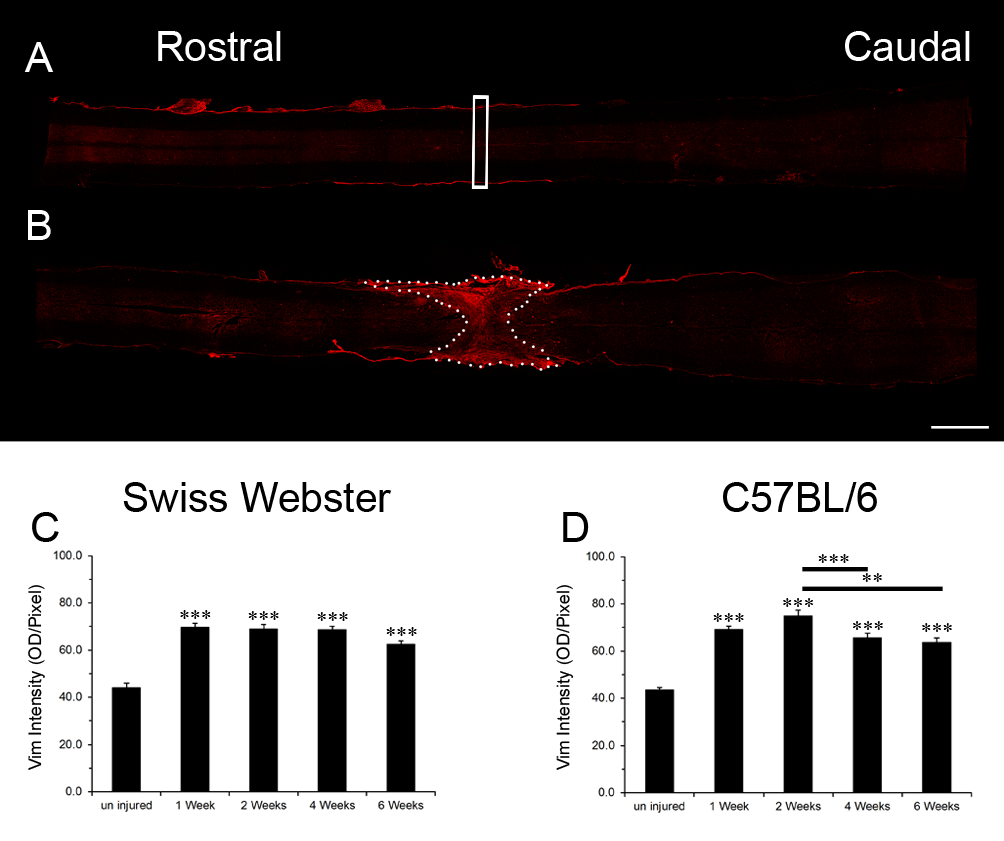

Supplement: FIGURE S4 — Astrogliosis within the lesion site in Swiss Webster and C57BL/6 mice after severe SCI. Confocal photomicrographs indicating vimentin staining in the NI control and transected longitudinal spinal cord sections (A,B). Bar graphs indicating quantitative analysis of vimentin immunoreactivity at different stages after spinal cord transection (C,D). Transected Swiss Webster and C57BL/6 mice displayed similar increase in vimentin immunoreactivity (C,D). One-way ANOVA with Tukey post hoc tests. **P < 0.01, ***P < 0.001. Scale bars (A,B): 1 mm. [file Image_4.TIF]
